# Matriptase-2 regulates iron homeostasis primarily by setting the basal levels of hepatic hepcidin expression through a nonproteolytic mechanism

**DOI:** 10.1016/j.jbc.2023.105238

**Published:** 2023-09-09

**Authors:** Caroline A. Enns, Tyler Weiskopf, Richard H. Zhang, Jeffrey Wu, Shall Jue, Makiko Kawaguchi, Hiroaki Kataoka, An-Sheng Zhang

**Affiliations:** 1 Department of Cell, Developmental, and Cancer Biology, Oregon Health & Science University, Portland, Oregon, USA; 2 Faculty of Medicine, Section of Oncopathology and Regenerative Biology, Department of Pathology, University of Miyazaki, Miyazaki, Japan

**Keywords:** hepatocyte, iron, homeostasis, hormone, serine protease, hepcidin, matriptase-2, hai-2

## Abstract

Matriptase-2 (MT2), encoded by *TMPRSS6*, is a membrane-anchored serine protease. It plays a key role in iron homeostasis by suppressing the iron-regulatory hormone, hepcidin. Lack of functional MT2 results in an inappropriately high hepcidin and iron-refractory iron-deficiency anemia. Mt2 cleaves multiple components of the hepcidin-induction pathway *in vitro*. It is inhibited by the membrane-anchored serine protease inhibitor, Hai-2. Earlier *in vivo* studies show that Mt2 can suppress hepcidin expression independently of its proteolytic activity. In this study, our data indicate that hepatic Mt2 was a limiting factor in suppressing hepcidin. Studies in *Tmprss6*^*−/−*^ mice revealed that increases in dietary iron to ∼0.5% were sufficient to overcome the high hepcidin barrier and to correct iron-deficiency anemia. Interestingly, the increased iron in *Tmprss6*^*−/−*^ mice was able to further upregulate hepcidin expression to a similar magnitude as in wild-type mice. These results suggest that a lack of Mt2 does not impact the iron induction of hepcidin. Additional studies of wild-type Mt2 and the proteolytic-dead form, fMt2^S762A^, indicated that the function of Mt2 is to lower the basal levels of hepcidin expression in a manner that primarily relies on its nonproteolytic role. This idea is supported by the studies in mice with the hepatocyte-specific ablation of Hai-2, which showed a marginal impact on iron homeostasis and no significant effects on iron regulation of hepcidin. Together, these observations suggest that the function of Mt2 is to set the basal levels of hepcidin expression and that this process is primarily accomplished through a nonproteolytic mechanism.

Matriptase-2 (MT2) is a type-II transmembrane serine protease. It is composed of a short N-terminal cytoplasmic domain, a transmembrane domain, and an extracellular domain of ∼735 amino acids, which contains a membrane-proximal stem region, a predicted activation domain, and a C-terminal catalytic domain (Fig. 1*A*) (1). MT2 is encoded by the *TMPRSS6* gene in humans and the *Tmprss6* gene in mice and is predominantly expressed in the liver (1). It plays a key role in iron homeostasis by suppressing hepatic *hepcidin* gene transcription (1, 2, 3, 4). Hepcidin is an iron-regulatory hormone that is secreted mainly by hepatocytes (3, 4). Hepcidin inhibits iron efflux from duodenal enterocytes that absorb dietary iron, from iron-recycling macrophages in the spleen and in the liver, and from iron-storing hepatocytes into the circulation by blocking the plasma-membrane iron exporter, ferroportin (FPN) (Fig. 1*B*) (5, 6). Lack of hepcidin causes juvenile hemochromatosis, a particularly severe iron overload disorder (7, 8). In humans, mutations in the *TMPRSS6* gene result in an inappropriately high hepcidin expression and cause iron-refractory iron-deficiency anemia (IRIDA) (3, 4, 9). Similar phenotypes and alopecia indicating low iron levels in the body are reported in mouse models with a global *Tmprss6* knockout (*Tmprss6*^*−/−*^), a truncated *Tmprss6* that lacks the coding sequence for the catalytic domain (*mask*), or an Ile286Phe substitution in the CUB domain (Fig. 1*A*) (10, 11, 12, 13, 14). Our earlier studies suggest that this Ile286Phe substitution in Mt2 reduces the interaction with its binding partners (15).

**Figure 1:**
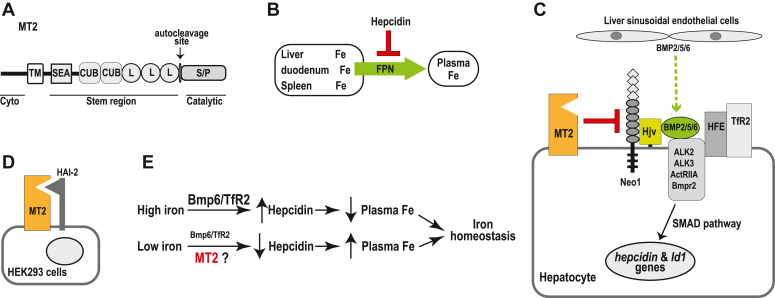
**Hepcidin is an iron regulatory hormone that is secreted mainly by hepatocytes.***A*, diagram of MT2 protein. Cyto, cytoplasmic domain; TM, transmembrane domain; SEA, sea urchin sperm protein, enteropeptidase agrin; CUB, complement protein subcomponents C1r/C1s, urchin embryonic growth factor and bone morphogenetic protein 1 domain; L, low-density lipoprotein receptor class-A domain; Catalytic, serine protease (S/P) catalytic domain. The *arrow* indicates the predicted autocleavage activation site. *B*, hepcidin inhibits iron efflux from duodenum, spleen, and liver into the circulation by blocking the plasma-membrane iron exporter, FPN. *C*, diagram of the key components that are involved in the induction and suppression of hepcidin expression in the liver. The inhibitor of the differentiation-1 gene (*Id1*) is a direct downstream target of BMP signaling. There is no known function of Id1 in iron homeostasis. *D*, diagram for HAI-2 inhibition of the proteolytic activity of MT2. *E*, iron induction of hepcidin expression by increases in *Bmp6* gene transcription and TfR2 protein stabilization.

Hepatic hepcidin expression is induced *via* the bone morphogenetic protein (BMP)-signaling pathway (16, 17, 18). BMP signaling is initiated upon the binding of BMP ligands to type-I and type-II BMP receptors. Hepatocytes utilize a selective set of BMP receptors, which include ALK2, ALK3, ActRIIA, and BMPR2, to induce hepcidin expression (19, 20). The essential Bmp ligands, Bmp2, Bmp5, and Bmp6, are derived from the liver sinusoidal endothelial cells adjacent to hepatocytes (21, 22, 23, 24, 25, 26, 27). Importantly, a normal range of hepcidin expression also requires other plasma membrane proteins including hemochromatosis protein (HFE), hemojuvelin (HJV), transferrin receptor-2 (TfR2), and neogenin (Neo1) (Fig. 1*C*) (3, 28, 29). In mice, global or hepatocyte-specific ablation of each of these genes reduces hepatic hepcidin expression and results in iron overload (28, 30, 31, 32, 33, 34). In humans, mutations in the *HFE*, *HJV*, or *TfR2* gene cause type-1, type-2A, and type-3 hemochromatosis, respectively (3, 8, 35).

MT2 is synthesized as a zymogen and undergoes activation by autocleavage on the cell surface (1, 36, 37). *In vitro* studies indicate that the activated MT2 cleaves multiple components of the hepcidin induction pathway, including HJV, Alk3, ActRIIA, Hfe, and Tfr2, and reduces their levels on the cell surface (Fig. 1*C*) (13, 38). Co-expression of the hepatocyte growth factor activator inhibitor-2 (Hai-2) inhibits the Mt2 proteolytic activity (Fig. 1*D*) (39, 40). Hai-2, encoded by the *Spint2* gene, is a broad membrane-associated serine protease inhibitor (39, 41). Initial *in vivo* studies suggest that MT2 suppresses hepcidin expression by inhibiting the function of HJV. The combined disruption of both *Hjv* and *Tmprss6* genes in mice displays a phenotype that is indistinguishable from *Hjv*-null (*Hjv*^*−/−*^) mice with a marked suppression of hepcidin expression and severe iron overload (12). Combined ablation of *Tmprss6* and *Hfe* or *Tfr2* genes shows a phenotype similar to the *Tmprss6*^*−/−*^ mice with an inappropriately high hepcidin expression and iron deficiency anemia (42, 43). Further studies imply that Mt2 can suppress hepcidin expression independently of Hjv because increased expression of exogenous Mt2 in the liver of *Hjv*^*−/−*^ mice is still able to significantly reduce hepcidin (13). MT2 can bind to HJV through its stem region (38). In *mask* mice that lack detectable Mt2 protein, the increased hepcidin expression is associated with a decrease, rather than an increase, in hepatic Hjv protein (44, 45). These findings support the idea that Mt2 suppression of hepcidin is not mediated through its proteolytic activity to cleave and to inactivate its substrates.

Mechanistic studies reveal that Mt2 suppression of hepcidin expression requires not only its catalytic domain but also the stem region (Fig. 1*A*) and that it has to be associated with the plasma membrane (40). This is consistent with the observations that the IRIDA-causing mutations in MT2 are found throughout the entire extracellular domain (14). Interestingly, our recent studies show that Mt2 is able to suppress hepatic hepcidin expression independently of its proteolytic activity and suggest that this process is likely accomplished through substrate association (15). Hepatic hepcidin expression is positively regulated by the levels of bodily iron load through the BMP-signaling pathway. The liver possesses an elegant but incompletely defined machinery to modulate the transcription of *hepcidin* gene in response to the changes in bodily iron status (3, 46, 47, 48). Hepatic Tfr2 is stabilized by iron-saturated holo-Tf (49, 50), and the transcription of *Bmp*6 gene in the liver sinusoidal endothelial cells is elevated by increased liver iron storage (16, 51). Tfr2 and Bmp6 are, therefore, thought to be the sensors for increased iron in circulation and in storage, respectively, to enhance hepcidin expression (Fig. 1*E*). MT2 is the major negative regulator of hepcidin expression in the liver (1, 2, 3, 4). However, the involvement of Mt2 in iron regulation of hepcidin and the extent to which the proteolytic activity is needed for the function of Mt2 in iron homeostasis is not clear.

In this study, we investigated the roles of Mt2 in iron regulation of hepcidin expression by using *Tmprss6*^−/−^ mice and hepatocyte-specific *Spint2* knockout (*Spint2*^*fl/fl*^*;Alb-Cre*^*+*^) mice. Results indicated that neither the proteolytic nor nonproteolytic function of Mt2 is involved in the iron regulation of hepcidin. Our data support the idea that Mt2 is a limiting factor in the suppression of hepcidin expression and that the major function of Mt2 is to set the basal levels of hepcidin expression.

## Results

### Mt2 is a limiting factor in the suppression of hepatic hepcidin expression

**Figure 2:**
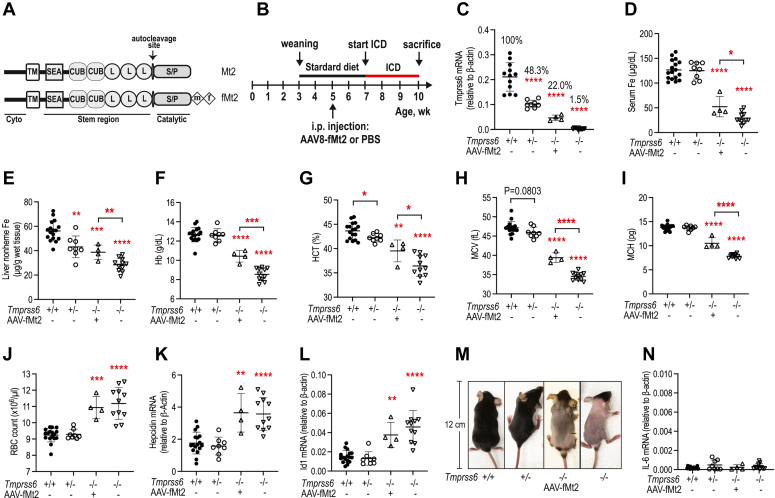
**Mt2 is a limiting factor in the suppression of hepatic hepcidin expression.***A*, diagrams of Mt2 and fMt2 constructs with C-terminal FLAG/MYC tag. m: MYC. f: FLAG. *B*, experimental design to determine the suppression of hepcidin expression relative to hepatic *Tmprss6* mRNA levels. 5-week-old homozygous *Tmprss6*^*−/−*^ mice were intraperitoneally injected with AAV8-fMt2 at ∼2 × 10^11^ viral genome particles per mouse or sterile PBS vehicle (−). Wild-type *Tmprss6*^*+/+*^ and heterozygous *Tmprss6*^*+/−*^ mice at the same age were also injected with vehicle. Standard diet (PicoLab Laboratory Rodent Diet-5L0D; LabDiet) contains 240-ppm iron. The iron control diet (ICD) contains 48 ppm iron. All mice were euthanized at ∼10 weeks of age for analysis. Each group consists of at least four mice with similar numbers of males and females. *C*–*L*, hepatic *Tmprss6* mRNA, serum iron, liver nonheme iron, hemoglobin (Hb), hematocrit (HCT), mean cell volume (MCV), mean corpuscular hemoglobin (MCH), RBC counts, hepatic *hepcidin* mRNA, and hepatic *Id1* mRNA levels in wild-type *Tmprss6*^*+/+*^ mice, heterozygous *Tmprss6*^*+/−*^ mice, as well as homozygous *Tmprss6*^*−/−*^ mice injected with AAV8-fMt2. *M*, a representative image of mice at the time of euthanasia. *N*, *IL-6* mRNA levels in the liver. All qRT-PCR results are expressed as the amount relative to that of β-actin for each sample. The mean values and the standard deviation (SD) are presented. One-way ANOVA was used to analyze the data relative to wild-type *Tmprss6*^*+/+*^ mice. Two-tailed student *t* test was also used to analyze the data between *Tmprss6*^*+/+*^ and *Tmprss6*^*+/−*^ mice as well as *Tmprss6*^*−/−*^ mice with and without AAV8-fMt2 administration. ∗*p* < 0.05; ∗∗*p* < 0.01; ∗∗∗*p* < 0.001; ∗∗∗∗*p* < 0.0001.

Earlier studies show that the heterozygous *Tmprss6*^+/−^ mice retain the ability to reduce hepcidin expression similar to the wild-type *Tmprss6*^+/+^ counterparts but they are more prone to iron deficiency when iron demands are high (12, 52). To determine the lowest levels of Mt2 that is needed to suppress hepcidin expression, we expressed FLAG/MYC-tagged Mt2 (fMt2; Fig. 2*A*) in the liver of *Tmprss6*^*−/−*^ mice at ∼22% *Tmprss6* mRNA of wild-type mice by intraperitoneal administration of AAV8 viral vector (Fig. 2, *B* and *C*). This AAV8 vector specifically expresses the gene of interest in hepatocytes, and the transduced gene is evenly expressed in hepatocytes throughout the liver (53), similar to the homogenous distribution of native *Tmprss6* mRNA in hepatocytes (21). Our earlier studies indicate that the addition of a C-terminal FLAG/MYC epitope to Mt2 did not affect its ability to suppress hepcidin expression (13, 15). In contrast to a full correction of the high hepcidin expression and iron deficient status by expressing a comparable *Tmprss6* mRNA level as in wild-type mice (13, 15), expression of ∼22% *Tmprss6* mRNA in *Tmprss6*^*−/−*^ mice was only able to moderately elevate serum iron concentration, liver nonheme iron, hemoglobin (Hb), hematocrit (HCT), mean cell volume (MCV), and mean corpuscular hemoglobin (MCH) with no evident change of red blood cell (RBC) counts (Fig. 2, *C*–*J*). There was no significant reduction of hepatic *hepcidin* and *Id1* mRNA levels and no evident improvement in alopecia (Fig. 2, *K*–*M*). The levels of liver nonheme iron are widely used as an indicator of iron storage in the body. *Id1* (inhibitor of differentiation 1) is a direct downstream target of BMP signaling (Fig. 1*C*), and the levels of *Id1* mRNA are widely used as a sensitive indicator for the status of BMP signaling. There is no known function of Id1 in iron homeostasis. In agreement with our earlier studies (54), the administration of the AAV8 viral vector did not increase *Il-6* mRNA (Fig. 2*N*), suggesting that no inflammation was induced. Consistent with the earlier observations (12, 52), *Tmprss6*^+/−^ mice at ∼10-week-old displayed a moderately lower level of liver nonheme iron and HCT (Fig. 2, *E* and *G*). A trend of lower MCV when compared with the corresponding wild-type counterparts (Fig. 2*H*) was also noted. These results suggest that at least 50% of *Tmprss6* mRNA seen in wild-type mice is required to efficiently suppress hepcidin expression. In conjunction with our earlier studies showing that increased Mt2 is able to further suppress hepcidin in wild-type and *Tmprss6*^*−/−*^ mice (13, 15), these observations indicate that Mt2 is a limiting factor in maintaining iron homeostasis.

### Lack of Mt2 does not significantly impact iron induction of hepatic hepcidin expression

**Figure 3:**
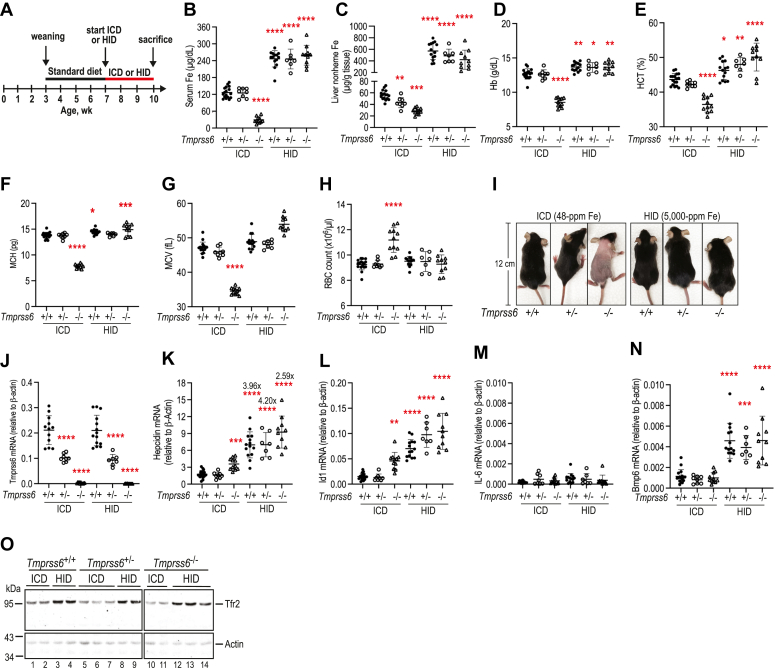
**Mt2 regulates iron homeostasis by setting the basal levels of hepatic hepcidin expression.***A*, experimental design to assess the iron induction of hepatic hepcidin expression. Seven-weeks old wild-type *Tmprss6*^*+/+*^, heterozygous *Tmprss6*^*+/−*^, and homozygous *Tmprss6*^*−/−*^ mice were randomly divided into two subgroups and fed either an iron control diet (ICD; 48 ppm iron) or a high iron diet (HID; 0.5% carbonyl iron). All mice were euthanized at ∼10 weeks of age for analysis. Each group consists of at least seven mice with similar numbers of male and female. *B*, serum iron assay. *C*, liver nonheme iron assay. *D*–*H*, blood parameters: Hb, HCT, MCH, MCV, and RBC counts. *I*, a representative image of mice at the time of euthanasia. Since the data for the ICD (48-ppm Fe) group were generated from the same sets of animal studies as in Figure 2, we used the same images for the ICD group as in Figure 2*M* in order to compare with the mice in the parallel HID (5000-ppm Fe) group. *J*–*N*, qRT-PCR analysis of hepatic *Tmprss6*, *hepcidin*, *Id1, IL-6,* and *Bmp6* mRNA levels. All qRT-PCR results are expressed as the amount relative to that of β-actin for each sample. *O*, representative images of Western blot analysis for endogenous Tfr2 and β-actin in the liver membrane extracts (250 μg protein) from the mice as described above. Each panel was cropped from the same image. All data are expressed as mean ± SD. One-way ANOVA and Tukey’s post-test were used to analyze the data relative to the *Tmprss6*^*+/+*^/ICD group. ∗*p* < 0.05; ∗∗*p* < 0.01; ∗∗∗*p* < 0.001; ∗∗∗∗*p* < 0.0001.

To seek insights into the roles of Mt2 in the iron regulation of hepcidin, we first tested the extent to which the *Tmprss6*^*−/−*^ mice can elevate hepatic hepcidin expression in response to increased bodily iron load. Previous studies indicate that severe iron deficiency in *mask* mice can be corrected by feeding an iron-enriched diet with 2% carbonyl iron (11, 45). To avoid gastrointestinal bleeding and other side effects of high dietary iron (55), we fed the *Tmprss6*^*+/+*^, *Tmprss6*^*+/−*^*,* and *Tmprss6*^*−/−*^ mice a high iron diet containing ∼0.5% carbonyl iron (HID). The parallel control groups were fed an iron control diet (ICD) containing 48 ppm iron. Since feeding a 0.83% carbonyl iron diet for 10 days is insufficient to fully correct the low Hb and low HCT status in *mask* mice (56), all animals were analyzed at 3 weeks after feeding the HID or ICD (Fig. 3*A*).

As expected, feeding a HID to wild-type mice (*Tmprss6*^*+/+*^/HID) markedly increased the bodily iron load as manifested by elevation of serum iron concentrations and liver nonheme iron levels (Fig. 3, *B* and *C*) and mild increases in Hb, HCT, and MCH (Fig. 3, *D*–*F*) when compared with the corresponding ICD group. Interestingly, feeding a HID to *Tmprss6*^*−/−*^ mice (*Tmprss6*^*−/−*^/HID) also led to a rapid iron load to a comparable level of *Tmprss6*^*+/+*^/HID group within 3 weeks (Fig. 3, *B* and *C*). As a result, the anemia was fully corrected as indicated by marked increases in Hb, HCT, MCH, and MCV (Fig. 3, *D*–*G*). The RBC counts returned to the level of *Tmprss6*^*+/+*^/ICD group (Fig. 3*H*), and the alopecia disappeared (Fig. 3*I*). In comparison, mice in *Tmprss6*^*−/−*^/ICD group remained severely iron deficient and anemic (Fig. 3, *B*–*I*). In wild-type mice, increased iron load did not alter hepatic *Tmprss6* mRNA levels (Fig. 3*J*). These observations indicate that the iron deficiency anemia in *Tmprss6*^*−/−*^ mice can be corrected by a diet containing ∼0.5% carbonyl iron.

Consistent with the earlier studies (3, 29, 57), increased bodily iron load significantly induced hepatic *hepcidin* expression in wild-type *Tmprss6*^*+/+*^ mice by facilitating the Bmp signaling as displayed by a parallel elevation in *Id1* mRNA (Fig. 3, *K* and *L*). Intriguingly, increased bodily iron load in *Tmprss6*^*−/−*^ mice was able to further upregulate both *hepcidin* and *Id1* mRNA levels to a similar magnitude as those seen in wild-type *Tmprss6*^*+/+*^ and heterozygous *Tmprss6*^*+/−*^ mice (Fig. 3, *K* and *L*). The relatively lower fold increases in *Tmprss6*^*−/−*^/HID group were likely due to the higher basal levels in the control *Tmprss6*^*−/−*^/ICD group (Fig. 3, *K* and *L*). The lack of significant change in *IL-6* mRNA levels (Fig. 3*M*) ruled out the possibility of inflammation. Thus, these observations indicate that lack of Mt2 does not significantly impact the iron-mediated upregulation of hepatic hepcidin expression. Because of the severe iron deficiency in *Tmprss6*^*−/−*^ mice fed an ICD (Fig. 3, *D*–*G*), no iron-deprivation studies were conducted in these animals.

We next examined whether the iron-induced hepcidin expression in *Tmprss6*^*−/−*^ mice resulted from elevated *Bmp6* mRNA and Tfr2 protein in the liver. Both Bmp6 and Tfr2 increase hepcidin expression (3, 29, 57). The transcription of *Bmp6* gene is upregulated by increased iron storage (16, 51), and hepatic Tfr2 is stabilized by increased iron-saturated holo-Tf (49, 50). As shown in Figure 3, *N* and *O*, similar extents of elevation in *Bmp6* mRNA and Tfr2 protein in the liver were detected between the *Tmprss6*^*+/+*^/HID and *Tmprss6*^*−/−*^/HID groups when compared with their corresponding ICD controls. Additionally, a comparable degree of increase was also detected in *Tmprss6*^*+/−*^ mice. These results indicate that *Tmprss6*^*−/−*^ mice retain intact iron-sensing machinery in the liver and that Mt2 is not involved in the iron regulation of Tfr2 stability and *Bmp6* gene transcription. Rather the abovementioned data suggest that the function of Mt2 is to set the basal level of hepcidin expression in the liver.

### The proteolytic activity of Mt2 is not required in iron induction of hepatic hepcidin expression

**Figure 4:**
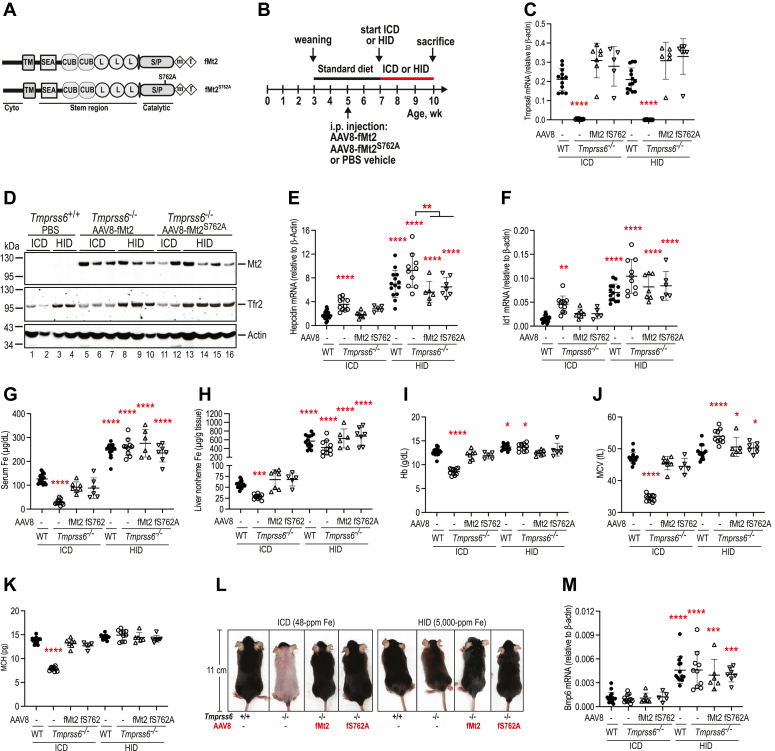
**Mt2 suppression of hepcidin expression largely depends on its nonproteolytic function.***A*, diagrams of wild-type fMt2 and the proteolytic dead fMt2^S762A^. *B*, experimental design to assess the role of Mt2 proteolytic activity in iron induction of hepcidin expression. Five-week-old *Tmprss6^−/−^* mice of both sexes were intraperitoneally injected with AAV8-fMt2 or fMt2^S762A^ at ∼8 × 10^11^ viral genome-particles per mouse, or the sterile PBS vehicle as control (−). At 2 weeks post the viral administrations, mice in each group were randomly divided into two subgroups and fed either an iron control diet (ICD; 48 ppm iron) or a high iron diet (HID; 0.5% carbonyl iron) for 3 weeks. Animals were euthanized at ∼10 weeks of age. Age-matched wild-type *Tmprss6^+/+^* (WT) littermates on the same background were injected with a sterile PBS vehicle as additional controls. Each group consists of at least five animals with similar numbers of male and female. *C*, qRT-PCR analysis of hepatic *Tmprss6* mRNA. *D*, representative images of Western blot analysis for transduced fMt2 and fMt2^S762A^, the endogenous Tfr2 and β-actin in the liver membrane extracts (250 μg protein) from the mice as described above. *E* and *F*, qRT-PCR analysis of hepatic *hepcidin* and *Id1* mRNA. *G*, serum iron assay. *H*, liver nonheme iron assay. *I*–*K*, Blood parameters: Hb, MCV, and MCH. *L*, a representative image of mice at the time of euthanasia. *M*, qRT-PCR analysis of hepatic *Bmp6* mRNA. All qRT-PCR results are expressed as the amount relative to that of β-actin for each sample. Data are expressed as mean ± SD. One-way ANOVA and Tukey’s post-test were used to analyze the data relative to the WT-*Tmprss6*^*+/+*^/ICD group. ∗*p* < 0.05; ∗∗*p* < 0.01; ∗∗∗*p* < 0.001; ∗∗∗∗*p* < 0.0001.

Our earlier studies indicate that Mt2 can suppress hepcidin expression through a nonproteolytic mechanism in mice fed a 240-ppm iron diet (15). To determine the roles of Mt2 proteolytic activity in iron homeostasis in response to dietary changes in iron, we compared the abilities of wild-type fMt2 and the proteolytic dead form, fMt2^S762A^, to suppress hepcidin expression under the control iron (48-ppm iron) and high iron (0.5% carbonyl iron) conditions. fMt2 and fMt2^S762A^ with a C-terminal FLAG/MYC epitope (Fig. 4*A*) were transduced into the liver of *Tmprss6*^−/−^ mice by AAV8 viral vectors as described in our earlier studies (15). AAV8-fMt2 and -fMt2^S762A^ viral vectors were intraperitoneally administered into the *Tmprss6*^*−/−*^ mice of both genders at ∼5 weeks of age. The *Tmprss6*^*+/+*^ and *Tmprss6*^−/−^ controls were injected with a sterile PBS vehicle. Our earlier studies demonstrated that the intraperitoneal administration of empty AAV8 vector had no evident impacts on hepatic *hepcidin* expression and iron homeostasis in mice (58, 59). At 2 weeks after viral administration to allow the expression of transduced Mt2 and Mt2^S762A^, animals were randomly divided into two categories to feed either an ICD (48-ppm iron) or a HID (0.5% carbonyl iron) for 3 more weeks (Fig. 4*B*). All mice were euthanized at ∼10 weeks old for analysis.

As shown in Figure 4*C*, the mRNA levels of transduced fMt2 and fMt2^S762A^ were modestly higher than those of the wild-type control group. Both fMt2 and fMt2^S762A^ proteins in the liver membrane preparations were readily detected by an anti-FLAG antibody (Fig. 4*D*). Consistent with our earlier studies (15), the expressed fMt2 and fMt2^S762A^ in the liver of *Tmprss6*^−/−^ mice exhibited comparable abilities to reduce the high *hepcidin* and *Id1* mRNA expression when fed an ICD. The *hepcidin* and *Id1* mRNA were decreased to a similar level of wild-type *Tmprss6*^+/+^/ICD group (Fig. 4, *E* and *F*). Consistently, both fMt2 and fMt2^S762A^ displayed similar extents of increases in serum iron concentrations, liver nonheme iron levels, and blood parameters including Hb, MCV, and MCH (Fig. 4, *G*–*K*). The alopecia also disappeared as a result of the increased bodily iron load (Fig. 4*L*). These observations indicate that the proteolytic activity of Mt2 is not required for the suppression of hepcidin expression in mice with an adequate iron supply.

In the HID groups, the fMt2-transduced *Tmprss6*^−/−^ mice displayed comparable levels of increases in serum iron and liver nonheme iron levels as seen in the wild-type and *Tmprss6*^−/−^ controls (Fig. 4, *G* and *H*). Increased bodily iron load resulted in similar extents of hepcidin and *Id*1 mRNA increases between fMt2/HID and wild-type *Tmprss6*^+/+^/HID groups (Fig. 4, *E* and *F*). These results indicate that the transduced fMt2 acts similarly to endogenously expressed Mt2 in response to a high iron load. Intriguingly, increased bodily iron in the fMt2^S762A^-transduced *Tmprss6*^−/−^ mice fed an HID (fMt2^S762A^/HID) also led to analogous increases in hepcidin and *Id*1 mRNA as seen in fMt2/HID and wild-type *Tmprss6*^+/+^/HID groups (Fig. 4, *E* and *F*). These observations indicate that the proteolytic activity of Mt2 is not involved in the iron induction of hepcidin expression. In comparison, the *Tmprss6*^−/−^/HID controls displayed a significantly higher level of hepcidin mRNA and a trend of increase in *Id1* mRNA (Fig. 4, *E* and *F*). The lack of changes of IL-6 mRNA (Fig. S1) ruled out the possibility of inflammation as a cause of elevated hepcidin expression. These data suggest that under the high iron conditions, Mt2 also acts as a suppressor to downshift the hepcidin expression. Additional analysis revealed that expression of neither fMt2 nor fMt2^S762A^ significantly affected the iron-mediated increases in hepatic Tfr2 protein and *Bmp6* mRNA (Fig. 4, *D* and *M*). These results imply that the Mt2 suppression of hepcidin is not accomplished by altering the hepatic Tfr2 levels or the *Bmp6* gene transcription. Taken together, the data suggest that the proteolytic activity of Mt2 is not involved in the iron induction of hepatic hepcidin expression and that the primary function of Mt2 is to downshift the basal level of hepcidin expression.

### Hepatocyte-specific ablation of the serine protease inhibitor, Hai-2, displays a marginal effect on hepcidin expression in mice

**Figure 5:**
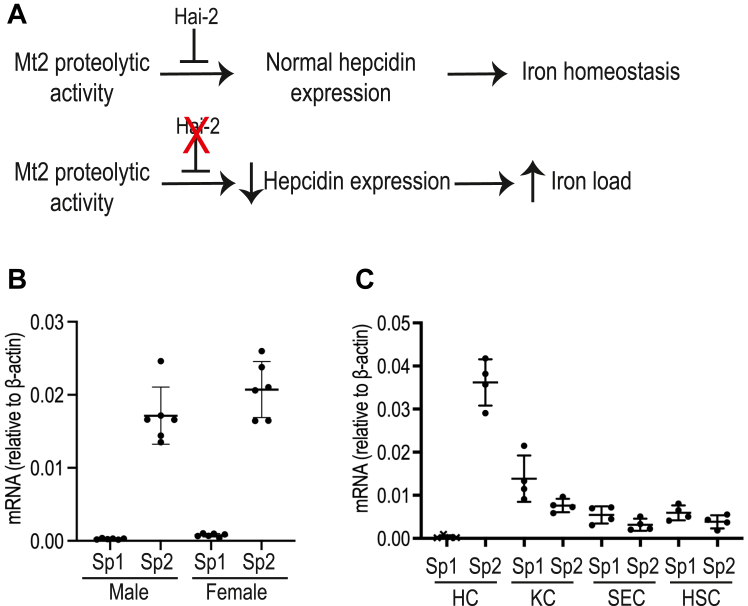
**Spint2, not Spint1, is predominantly expressed in hepatocyte population of the liver.***A*, the predicted role of hepatic Hai-2 in Mt2 suppression of hepcidin expression and the predicted consequence of hepatic *Spint2* gene knockout in mice. *B*, qRT-PCR analysis of *Spint1* (Sp1) and *Spint2* (Sp2) mRNA levels in the liver of 8-week-old wild-type mice on a C57BL/6J background. Each group consists of at least five animals. *C*, qRT-PCR analysis of *Spint1* (Sp1) and *Spint2* (Sp2) mRNA levels in isolated hepatocytes (HC), Kupffer cells (KC), sinusoidal endothelial cells (SEC), and hepatic stellate cells (HSC) from the liver of wild-type 129S mice (n = 4). All qRT-PCR results are expressed as the amount relative to that of β-actin for each sample.

HAI-1 (encoded by the *Spint1* gene) and HAI-2 (encoded by the *Spint2* gene) are two closely related membrane-associated Kunitz-type serine protease inhibitors with a broad inhibitory spectrum (39, 60, 61, 62, 63, 64, 65, 66, 67). In transfected cells, Hai-2 blocks Mt2 cleavage of multiple key components in the hepcidin induction pathway (15, 39, 40, 60). We hypothesized that the lack of difference between fMt2 and the protease-dead fMt2^S762A^ in suppressing hepcidin expression could result from a constant inhibition of Mt2 proteolytic activity by the endogenously expressed Hai-2 in the liver (Fig. 5*A*). To test this hypothesis, we examined the expression profiles of Hai-1 and Hai-2 in the liver. qRT-PCR analysis revealed that the liver predominantly expresses *Spint-2* mRNA (Fig. 5*B*). In isolated cells from the mouse liver, Hai-2 mRNA was detected primarily in the hepatocyte population (Fig. 5*C*), which is similar to the expression profile of *Tmprss6* mRNA (21). Thus, both Hai-2 and Mt2 are co-expressed in hepatocytes.

**Figure 6:**
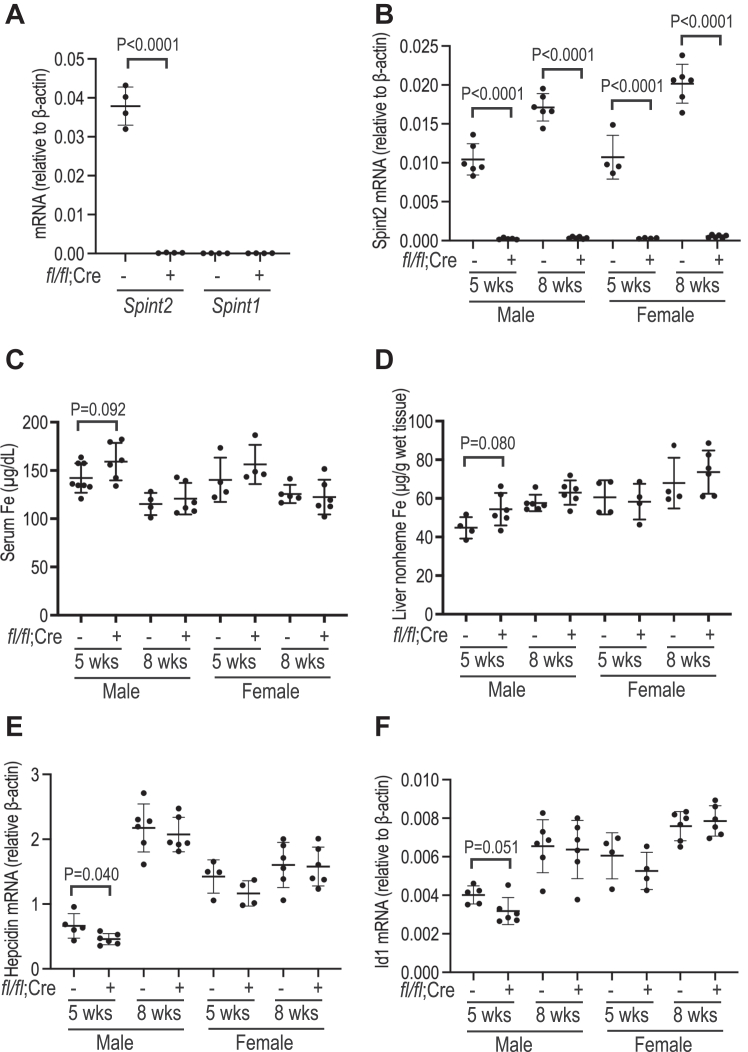
**Ablation of hepatic Spint2 mildly increases bodily iron load in male mice.***A*, qRT-PCR analysis of *Spint1* and *Spint2* mRNA levels in isolated hepatocytes from 8-week-old *Spint2*^*fl/fl*^*;Alb-Cre*^*-*^ and *Spint2*^*fl/fl*^*;Alb-Cre*^*+*^ mice. *B*, qRT-PCR analysis of *Spint2* mRNA levels in the liver of 5 and 8-week-old *Spint2*^*fl/fl*^*;Alb-Cre*^*-*^ and *Spint2*^*fl/fl*^*;Alb-Cre*^*+*^ mice of both genders. *C* and *D*, serum iron and liver nonheme iron assays in 5 and 8-week-old *Spint2*^*fl/fl*^*;Alb-Cre*^*-*^ and *Spint2*^*fl/fl*^*;Alb-Cre*^*+*^ mice of both genders. *E* and *F*, qRT-PCR analysis of *hepcidin* and *Id1* mRNA levels in the liver of 5 and 8-week-old *Spint2*^*fl/fl*^*;Alb-Cre*^*-*^ and *Spint2*^*fl/fl*^*;Alb-Cre*^*+*^ mice of both genders. All qRT-PCR results are expressed as the amount relative to that of β-actin for each sample. Each group consists of at least four animals. The means ± SD are presented. Two-tailed Student *t* test was used to analyze the data for each age and gender-matched group. The *p* values were presented.

To determine the roles of hepatic Hai-2 in hepcidin expression, we generated a liver-specific *Spint2* knockout (*Spint2*^fl/fl^;*Alb-Cre*^+^) mouse model by crossing *Spint2*^fl/fl^ mice (68) with mice that express a *Cre* recombinase transgene driven by a hepatocyte-specific albumin (*Alb*) promoter. Global ablation of *Spint2* gene in mice results in an embryonic lethality (69). qRT-PCR analysis revealed ∼99% depletion of *Spint2* mRNA in the isolated hepatocytes from *Spint2*^fl/fl^;*Alb-Cre*^+^ mice (Fig. 6*A*), indicating a hepatocyte-specific deletion. Distinct from global *Spint2* mutant mice, *Spint2*^fl/fl^;*Alb-Cre*^+^ mice were born in the predicted Mendelian ratio. There was no evident difference in appearance when compared to wild-type *Spint2*^*fl/fl*^*;Alb-Cre*^*-*^ littermates. Both *Spint2*^*fl/fl*^*;Alb-Cre*^*+*^ and *Spint2*^*fl/fl*^*;Alb-Cre*^*-*^ littermates had similar gender-dependent body weights at 5- and 8 weeks old (Fig. S2*A*). Both male and female *Spint2*^*fl/fl*^*;Alb-Cre*^*+*^ mice were fertile. Depletion of *Spint2* mRNA did not cause a compensatory increase of *Spint1* mRNA (Fig. 6*A*) and did not alter the mRNA levels of other key iron regulatory genes, including *Tmprss6, Hjv, Hfe, Tfr2,* or *Neo1*, in the isolated hepatocytes (Fig. S2, *B*–*F*).

The roles of endogenous Hai-2 in iron homeostasis were examined by comparing 5- and 8-week-old *Spint2*^*fl/fl*^*;Alb-Cre*^*+*^ mice with the gender and age-matched wild-type *Spint2*^*fl/fl*^*;Alb-Cre*^*-*^ littermates. All mice were fed a regular rodent diet containing 240-ppm iron after weaning. Consistent with the results in isolated hepatocytes (Fig. 6*A*), there were negligible amounts of *Spint2* mRNA in the liver of *Spint2*^*fl/fl*^*;Alb-Cre*^*+*^ mice (Fig. 6*B*). Compared with the corresponding wild-type *Spint2*^fl/fl^;*Alb-Cre*^-^ littermates, we detected a trend increase in serum iron and liver nonheme iron levels only in 5-week-old male *Spint2*^*fl/fl*^*;Alb-Cre*^*+*^ mice (Fig. 6, *C* and *D*). qRT-PCR analysis revealed a minor reduction in hepatic *hepcidin* and *Id1* mRNA levels in this group of *Spint2*^*fl/fl*^*;Alb-Cre*^*+*^ mice (Fig. 6, *E* and *F*). These results suggest that the moderate elevation of iron load in 5-week-old male *Spint2*^*fl/fl*^*;Alb-Cre*^*+*^ mice likely results from the reduced Bmp signaling and hepcidin expression. No evident difference was observed in other groups (Fig. 6, *C*–*F*). Ablation of the hepatic *Spint2* gene did not significantly alter the blood parameters, including RBC counts, Hb, HCT, MCV, and MCH in *Spint2*^*fl/fl*^*;Alb-Cre*^*+*^ mice (Table S1). Together, these results suggest that hepatic Hai-2, to a very limited extent, is involved in preventing the excessive suppression of hepatic hepcidin expression under steady state conditions.

### Lack of hepatic Hai-2 does not alter iron regulation of hepcidin expression

**Figure 7:**
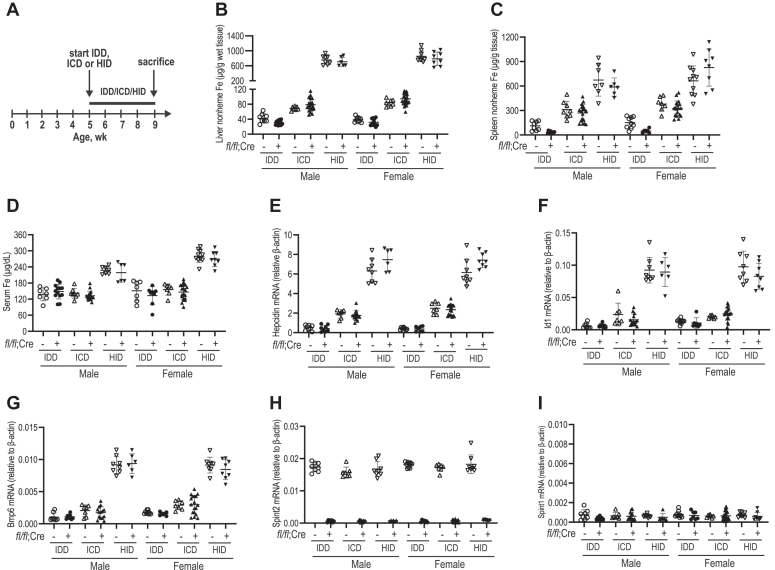
**Ablation of hepatic Spint2 does not affect iron regulation of hepcidin expression.***A*, experimental design to examine the effects of hepatocyte-specific *Spint2* knockout on iron regulation of hepcidin expression. Five-week-old *Spint2*^*fl/fl*^*;Alb-Cre*^*-*^ and *Spint2*^*fl/fl*^*;Alb-Cre*^*+*^ mice of both genders were fed an iron deficient diet (IDD; 2–6 ppm iron), an iron control died (ICD; 48 ppm iron), or a high iron diet (HID; 0.5% carbonyl iron; TD.140464) for 4 weeks before euthanasia for analysis. *B*–*D*, liver nonheme iron, spleen nonheme, and serum iron assays. *E*–*I*, qRT-PCR analysis of *hepcidin, Id1, Bmp6, Spint2,* and *Spint1* mRNA levels. All qRT-PCR results are expressed as the amount relative to that of β-actin for each sample. Each group consists of at least six animals. The means ± SD are presented. Two-tailed Student *t* test was used to analyze the data for each gender and diet-matched group. No statistical significance was noticed.

We next examined whether hepatic Hai-2 is required for iron regulation of hepcidin expression. Both *Spint2*^*fl/fl*^*;Alb-Cre*^*+*^ and the wild-type *Spint2*^*fl/fl*^*;Alb-Cre*^*-*^ littermates of both genders were fed an iron-deficient diet (IDD; 2–6 ppm iron), an ICD (48 ppm iron), or a HID (0.5% carbonyl iron) for 3 weeks before analysis (Fig. 7*A*). As shown in Figure 7, *B*–*D*, mice fed a HID displayed marked increases in the liver and spleen nonheme iron levels as well as serum iron concentrations in both *Spint2*^*fl/fl*^*;Alb-Cre*^*+*^ and *Spint2*^*fl/fl*^*;Alb-Cre*^*-*^ mice to the similar extents. Consistently, comparable decreases in the liver and spleen nonheme iron were also detected between the IDD groups (Fig. 7, *B* and *C*). Although mice fed an IDD displayed no significant reduction of serum iron levels relative to their corresponding ICD controls (Fig. 7*D*), all these mice had a trend toward iron deficiency anemia as manifested by mild decreases in Hb, HCT, MCV, or MCH (Table S2). These observations indicate that depletion of hepatic Hai-2 does not significantly impact the bodily iron loading in mice.

As expected, hepatic *hepcidin*, *Id1*, and *Bmp6* mRNA levels in wild-type *Spint2*^*fl/fl*^*;Alb-Cre*^*-*^ mice of both genders were decreased by bodily iron load deprivation and elevated by increased body iron load (Fig. 7, *E*–*G*). These observations are consistent with the idea that hepcidin is induced *via* the Bmp signaling and that *Bmp6* expression in the liver is enhanced by increased liver iron load. In comparison, increased iron load in *Spint2*^*fl/fl*^*;Alb-Cre*^*+*^/HID groups displayed similar extents of enhancement in hepatic *hepcidin*, *Id1*, and *Bmp6* mRNA as seen in the corresponding *Spint2*^*fl/fl*^*;Alb-Cre*^*-*^/HID controls (Fig. 7, *E*–*G*). Consistently, similar extents of decrease in hepatic *hepcidin*, *Id1*, and *Bmp6* mRNA were observed by iron depletion between the Hai-2-ablated *Spint2*^*fl/fl*^*;Alb-Cre*^*+*^ mice (*Spint2*^*fl/fl*^*;Alb-Cre*^*+*^/IDD) and wild-type *Spint2*^*fl/fl*^*;Alb-Cre*^*-*^ mice (*Spint2*^*fl/fl*^*;Alb-Cre*^*-*^/IDD) of both genders (Fig. 7, *E*–*G*). The lack of change of the *Spint2* or *Spint1* mRNA levels by different bodily iron load (Fig. 7, *H* and *I*) suggests that their expressions are not regulated by the Bmp signaling. Additionally, we also examined the induction of hepcidin expression by acute iron increase in *Spint2*^*fl/fl*^*;Alb-Cre*^*+*^ mice. No significant difference was detected when compared with *Spint2*^*fl/fl*^*;Alb-Cre*^*-*^ mice (data not shown). Together, these observations indicate that lack of hepatic Hai-2 does not impact the iron regulation of hepcidin expression, and suggest that hepatic Hai-2 does not play an important role in Mt2 suppression of hepcidin.

## Discussion

In this study, we explored the underlying mechanism by which hepatic Mt2 suppresses hepcidin expression. Our results indicate that hepatic Mt2 was a limiting factor in iron homeostasis, and that lack of Mt2 did not impact the magnitude of hepcidin induction by iron. Further studies suggest that the major function of Mt2 was to set the basal levels of hepatic hepcidin expression and that this process was accomplished primarily by its nonproteolytic function. Consistently, ablation of hepatocyte Hai-2 in mice only displayed a marginal effect on iron homeostasis. In conjunction with the earlier studies (15), our data support the idea that Mt2 regulates iron homeostasis primarily by setting the basal levels of hepatic hepcidin *via* a nonproteolytic mechanism.

Earlier studies show that the heterozygous *Tmprss6*^+/−^ mice develop normally and are phenotypically indistinguishable from wild-type littermates (52). They have similar abilities to reduce hepcidin expression as the wild-type counterparts but are prone to iron deficiency when iron demands are high or when dietary iron is restricted (12, 52). Here, we explored the minimal levels of Mt2 that are needed to suppress hepcidin expression. Results indicate that the expression of ∼22% *Tmprss6* mRNA of wild-type mice in *Tmprss6*^*−/−*^ mice was unable to correct the high hepcidin expression and iron deficiency status. Interestingly, the siRNA and antisense oligonucleotide knockdown studies in wild-type mice reveal a significant increase in hepcidin expression when hepatic *Tmprss6* mRNA levels were decreased by ∼60% (70, 71). This increase in hepcidin expression was also augmented by further reduction of *Tmprss6* mRNA (70, 71). These observations suggest that an efficient suppression of hepatic hepcidin expression requires at least 50% of *Tmprss6* mRNA seen in wild-type mice. Conversely, our earlier studies show that increased Mt2 in wild-type mice is able to further suppress hepcidin mRNA levels and to increase serum iron concentrations (13). Since MT2 expression is not transcriptionally regulated by iron (21, 72), these findings indicate that hepcidin expression is negatively correlated with hepatic *Tmprss6* mRNA levels, and support the idea that Mt2 is a limiting factor in the suppression of hepcidin.

The liver possesses an elegant but incompletely defined machinery that can sense the levels of bodily iron load and positively regulate the transcription of the *hepcidin* gene (3, 46, 47, 48, 57). This iron sensing machinery appears to be altered by the *TMPRSS6* gene mutations in humans (3, 4, 9), global ablation of *Tmprss6* gene (*Tmprss6*^*−/−*^) or a deletion of the Mt2 catalytic domain (*mask*) in mice (10, 11, 12, 13), because they all result in an inappropriately high hepcidin and iron-deficiency anemia. Here we investigated the roles of Mt2 in iron regulation of hepcidin expression. First, our data validated the earlier observations that iron-deficiency anemia in *mask* mice could be corrected by feeding a rodent diet with 0.83% or 2% carbonyl iron (11, 45, 56). We found that feeding a 0.5% carbonyl iron diet in *Tmprss6*^*−/−*^ mice was sufficient to overcome the high hepcidin barrier and to load iron into the liver to a similar extent as seen in *Tmprss6*^*+/+*^ and *Tmprss6*^*+/−*^ mice within 3 weeks. The lower “high iron diet” minimized the deleterious side effects of damage to the intestines and kidneys. Interestingly, when the response of hepatic hepcidin expression to increased bodily iron load was compared, our data revealed that the *Tmprss6*^*−/−*^ mice retained the comparable responses of wild-type *Tmprss6*^*+/+*^ and heterozygous *Tmprss6*^*+/−*^ mice to elevate hepatic *hepcidin* expression by enhancing the Bmp signaling. The absolute levels of hepcidin mRNA in the *Tmprss6*^*−/−*^ mice remained higher than those seen in *Tmprss6*^*+/+*^ and *Tmprss6*^*+/−*^ mice, similar to the ICD groups. Further studies indicated that lack of Mt2 did not impact the increases in hepatic Tfr2 protein and *Bmp6* mRNA by increased iron load in *Tmprss6*^*−/−*^ mice. Hepatic Tfr2 is stabilized by increased iron-saturated holo-Tf in the circulation (49, 50), and the transcription of *Bmp6* gene in the liver sinusoidal endothelial cells is upregulated by an increased intracellular iron (16, 51, 73). These results suggest that *Tmprss6*^*−/−*^ mice have intact iron-sensing and response machinery in the liver. Together, these observations support the idea that the function of Mt2 is to set the basal levels of hepcidin expression in the liver.

MT2 is a membrane-anchored serine protease. It cleaves multiple components of the hepcidin induction pathway including Tfr2 and Hjv, and reduces their levels on the cell surface in transfected cells (13, 38). Our earlier *in vivo* studies indicate that Mt2 can suppress hepcidin expression independently of its proteolytic activity in mice under the steady state conditions (15). Here we investigated the role of proteolytic activity of Mt2 in iron regulation of hepcidin expression by taking advantage of fMt2 and the protease-dead fMt2^S762A^. Our data indicate that the protease-dead fMt2^S762A^ behaved similarly to wild-type fMt2 in the suppression of hepatic hepcidin expression and the increase of bodily iron load under both adequate and high iron conditions. Consistently, expression of neither wild-type Mt2 nor fMt2^S762A^ impacted the iron-induced increases in hepatic Tfr2 protein. These observations suggested that the proteolytic activity of Mt2 is not involved in the iron induction of hepcidin expression. In support of this idea, the decreased, rather than increased, levels of Hjv protein are found in the liver of *mask* mice that lack the Mt2 catalytic domain (44). Our earlier studies also suggest that the levels of hepatic Hjv is not a limiting factor in the induction of hepcidin (54, 58). Thus, Mt2 suppression of hepcidin expression was unlikely mediated through its proteolytic activity to cleave and to inactivate Hjv and other hepcidin-inducing components. Rather, these findings favor our earlier hypothesis that Mt2 suppress hepcidin expression through the interaction with its binding partners (15).

Additional studies in mice with hepatocyte-specific ablation of Hai-2 strengthen the idea that Mt2 suppresses hepcidin expression independent of its proteolytic activity. HAI-2 and HAI-1 are two closely related membrane-associated Kunitz-type serine protease inhibitors with a broad inhibitory spectrum, including MT2, matriptase, hepsin (also known as Tmprss1), and prostasin (39, 60, 61, 62, 63, 64, 65, 66, 67, 74, 75). Matriptase is a close family member of MT2 (1). Other studies indicate that the function of matriptase *in vivo* is controlled by HAI-2. Lack of Hai-2 suppression of the matriptase-mediated cell surface proteolysis causes neural developmental defects (76), prostate cancer invasion (77, 78, 79, 80), and severe intestinal epithelial damage (68, 81). We found that the hepatocytes predominantly expressed Hai-2, but not Hai-1, which is similar to that of Mt2 (21). Our earlier studies showed that marked increases of Hai-2 in the liver of wild-type mice did not significantly affect hepcidin expression and Bmp signaling (15). This observation could be interpreted to mean that the endogenously expressed Hai-2 is sufficient to inhibit the proteolytic activity of Mt2. In this study, we detected a mild decrease in hepatic hepcidin mRNA levels and moderate increases in serum iron and liver nonheme iron only in 5-week-old male mice with hepatocyte-specific Hai-2 ablation. This decrease in hepcidin expression could result from the lack of Hai-2 to inhibit the Mt2 proteolytic activity. Alternatively, this decrease in hepcidin expression could also be caused by the lack of Hai-2 to interfere with the non-proteolytic function of Mt2. *In vitro* studies show that HAI-2 binds to and forms a complex with MT2 (39). Additionally, it is also possible that this decreased hepcidin expression is caused by altered functions of hepatic hepsin and prostasin. Both hepsin and prostasin are expressed in the liver, and they are essential for various liver metabolism and energy homeostasis (82, 83, 84). Further studies indicate that lack of hepatic Hai-2 did not significantly impact the iron regulation of hepcidin in mice. These data strengthened the idea that the proteolytic activity of Mt2 is not involved in iron regulation of hepcidin. Together, these observations suggest that hepatic Hai-2 might play a minor role in iron homeostasis by increasing hepcidin expression in young mice. We speculate that this process is likely accomplished by Hai-2-mediated inhibition of the proteolytic activity or non-proteolytic function of Mt2.

**Figure 8:**
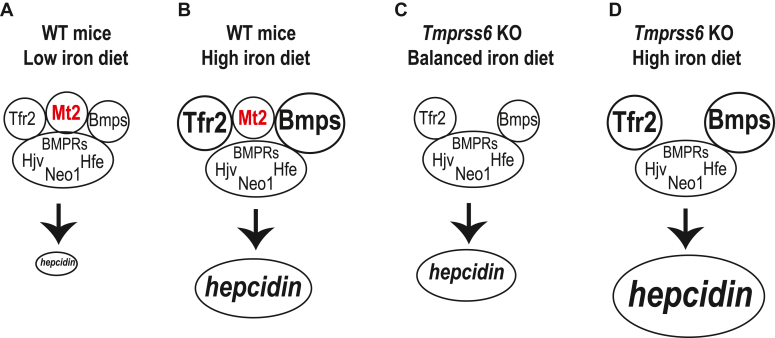
**A model for Mt2 suppression of hepcidin expression through its nonproteolytic activity.** In wild-type mice, Mt2 inhibits hepcidin expression primarily by binding to and interfering with the hepcidin-inducing complex that contains BMP receptors (BMPRs), Hjv, Tfr2, Hfe, and Neo1 on the plasma membrane of hepatocytes. The iron induction of hepcidin expression is accomplished by holo-Tf-mediated stabilization of Tfr2 and the upregulated transcription of *Bmp6* gene in the liver sinusoidal endothelial cells by increased iron store (*A* and *B*). Lack of Mt2 will upper-shift the hepcidin expression with no impact on iron-mediated induction (*C* and *D*).

In summary, our data support the idea that Mt2 regulates iron homeostasis by setting the basal levels of hepatic *hepcidin* gene expression *via* a nonproteolytic mechanism. Based on the findings in this and previous studies showing that the increased hepcidin expression in *mask* mice is associated with a decrease, rather than increase, in hepatic Hjv protein (44, 45) and that at least three disease-causing mutations in human MT2 have unaltered proteolytic activity (85, 86, 87), we propose a model in which Mt2 inhibits hepcidin expression primarily by binding to and interfering with the complex in hepatocytes that facilitates the Bmp signaling, which leads to a downshift in the Bmp signaling and hepcidin expression to a constant extent (Fig. 8). In this scenario, the iron induction of hepcidin is accomplished by hepatic Tfr2 that is stabilized by iron-saturated holo-Tf in the circulation (49, 50) and Bmp6 in the liver sinusoidal endothelial cells whose transcription is upregulated by increased iron store (16, 51, 73). Mt2 is not involved in the iron regulation of hepcidin expression. The proteolytic activity of Mt2 may play a modest role in iron homeostasis at younger age when iron demands are high.

## Experimental procedures

### cDNA constructs

We purchased mouse Mt2 ORF (NM_027902.1) with a C-terminal FLAG/MYC epitope in pCMV6 vector from OriGene. We generated pCMV6-Mt2^S762A^, AAV8-Mt2, and AAV8-Mt2^S762A^ constructs in our previous studies (15). AAV8-Mt2 and Mt2^S762A^ vectors were generated at the Molecular Virology Support Core, OHSU.

### Animal studies

All animal procedures were approved by OHSU DCM. We purchased heterozygous *Tmprss6*^*+/−*^ mutant mice on S129/C57BL/6J background (13) and generated heterozygous *Tmprss6*^*+/−*^ mutant mice on C57BL/6J background after backcrossing with C57BL/6J mice for eleven generations. Heterozygous *Tmprss6*^*+/−*^, homozygous *Tmprss6*^*−/−*^, and wild-type *Tmprss6*^*+/+*^ mice were generated by breeding *Tmprss6*^*+/−*^ mice on C57BL/6J background.

To study the underlying mechanism by which Mt2 suppresses hepcidin, five-week-old *Tmprss6*^*−/−*^ mice of both genders were intraperitoneally injected with AAV8-Mt2 or AAV8-Mt2^S762A^ viral vectors at the doses indicated in the figure legends. Since our earlier studies demonstrated that an intraperitoneal administration of an empty AAV8 vector had no evident impacts on hepatic *hepcidin* expression and iron homeostasis in mice (58, 59), the control groups were injected with sterile PBS vehicle. At 2 weeks after the viral administrations, animals were randomly divided into two groups and started to be fed an ICD (48 ppm iron; TD.09488) or a HID (0.5% carbonyl iron; TD.140464) for an additional 3 weeks. Mice were euthanized at around 10 weeks old for analysis (Figs. 2*B* and 4*B*). Age, gender, and background-matched wild-type littermates were included as additional controls.

To study the roles of Mt2 in iron regulation of hepcidin expression, 7-week-old *Tmprss6*^*+/+*^, *Tmprss6*^*+/−*^, and *Tmprss6*^*−/−*^ mice of both genders were randomly divided into two groups and fed either an iron control diet (ICD; 48 ppm iron) or a high iron diet (HID; 0.5% carbonyl iron) for 3 weeks. Mice were euthanized at around 10 weeks old for analysis (Fig. 3*A*).

The *Spint2*-floxed (*Spint2*^fl/fl^) mice on a C57BL/6J background were generated as previously described (68). Homozygous hepatocyte-specific conditional *Spint2* knockout (*Spint2*^*fl/fl*^*;Alb-Cre*^*+*^) mice and littermate *Cre*^*–*^ controls were generated by crossing *Spint2*^*flflt*^ mice with mice expressing a *Cre* recombinase transgene driven by a hepatocyte-specific albumin (*Alb*) promoter on a C57BL/6J background (Jackson Laboratory). Genotyping was performed by using mouse tail snipping and PCR. The mice of both genders were euthanized at about 5 and 8 weeks of age for analysis. To study the roles of Hai-2 in iron regulation of hepcidin expression, six-week-old *Spint2*^*fl/fl*^*;Alb-Cre*^*+*^ and *Spint2*^*fl/fl*^*;Alb-Cre*^*-*^ mice of male or female were randomly divided into three groups and fed an iron-deficient diet (IDD; 2–6 ppm iron; TD.110669), an iron control diet (ICD; 48 ppm iron) or a HID (0.5% carbonyl iron) for 3 weeks. Mice were euthanized at around 9 weeks old for analysis (Fig. 7*A*).

To study the induction of hepcidin expression by acute iron increase, six-week-old *Spint2*^*fl/fl*^*;Alb-Cre*^*+*^ and *Spint2*^*fl/fl*^*;Alb-Cre*^*-*^ mice were first fed an iron-deficient diet (IDD; 2–6 ppm iron; TD.110669) for 3 week to deplete bodily iron. Animals were then randomly divided into two categories and intraperitoneally injected with either Venofer (American Regant, Inc #NOC 0517-2340-10) at ∼2 mg Fe per 100 g body weight or 30% sucrose/saline vehicle. At ∼4 h post-administration, mice were euthanized for analysis.

### Isolation of hepatocytes

Hepatocytes were isolated from 8-week-old *Spint2*^*fl/fl*^*;Alb-Cre*^*+*^ and *Spint2*^*fl/fl*^*;Alb-Cre*^*-*^ mice as previously described (51). Briefly, the livers were perfused using collagenase Type 2 (Worthington Biochemical Corporation) in Earle’s Balanced Salt Solution (Sigma-Aldrich). Hepatocytes were pelleted by centrifugation (500 rpm) (Beckman Centrifuge, Allegra 6R) for 5 min at 4 °C. Cell pellets were immediately lysed in the RA1 buffer of NucleoSpin RNAII kit for RNA preparation and qRT-PCR analysis of the genes of interest.

### Blood parameters and serum iron assay

Blood parameters were analyzed by using Hemavet 950 (Drew Scientific). Serum iron concentration was detected by using a Pointe Iron/TIBC Reagent Set (Pointe Scientific).

### Tissue nonheme iron assays

Tissue nonheme iron levels were determined as previously described (88) with the following modifications. Briefly, 50 to 150 mg wet tissues were digested in 250 to 750 μl of acid buffer at 65 °C for 72 h. The supernatant was collected by centrifugation at 10,000*g* for 5 min, followed by the addition of chromogen (1.86 mM bathophenanthroline sulfonate, 143 mM thioglycolic acid in water) and OD measurement at 535 nm. Each sample was measured twice in triplicate. Iron concentration is expressed as micrograms of iron per gram of wet tissue.

### qRT-PCR

Total RNA from mouse liver tissues and isolated hepatocytes was extracted using a NucleoSpin RNA kit (Macherey-Nagel). cDNA was synthesized using Oligo deoxythymidine primers (Invitrogen) and M-MLV reverse transcriptase (Invitrogen). The cDNA preparations from isolated mouse liver hepatocytes, Kupffer cells, sinusoidal endothelial cells, and hepatic stellate cells (HSC) are same as in our previous studies (89). qRT-PCR analysis was carried out in triplicate on each sample using the Power SYBR Green PCR master mix in a QuantStudio 12K Flex qPCR System (ThermoFisher). All primer sets used in these studies (Tables S3) were validated against the reference primers (β-actin) to ensure approximately equal efficiencies of amplification. The results are expressed as the amount relative to that of β-actin for each sample.

### Immunodetection

Liver membrane fractions were prepared as previously described (44). Protein extracts from the liver membrane preparations were separated by using SDS-PAGE under reducing conditions. The transduced exogenous fMt2 and fMt2^S762A^ in the liver were probed directly by using an HRP-coupled mouse anti-FLAG M2 IgG (Sigma) and chemiluminescence. The endogenous Tfr2 and β-actin were detected by using purified rabbit anti-Tfr2 (90), mouse anti-β-actin (Sigma), and the corresponding secondary antibodies.

### Statistical analysis

Two-tailed student *t* test was used to compare two sets of data. One-way ANOVA and Tukey’s post-test were used for multiple comparisons.

## Data availability

Raw data are available from the corresponding author on reasonable request.

## Supporting information

This article contains supporting information.

## Conflict of interest

The authors declare that they have no conflicts of interest with the contents of this article.
