# Supplementary material for: Matriptase-2 regulates iron homeostasis primarily by setting the basal levels of hepatic hepcidin expression through a nonproteolytic mechanism
Source: J Biol Chem. 2023 Sep 9;299(10):105238. doi: 10.1016/j.jbc.2023.105238 (PMC10551898; doi:10.1016/j.jbc.2023.105238)
Supplement: Supporting Tables S1–S3 and Figures S1 and S2 [file mmc1.pdf]

Matriptase-2 Regulates Iron Homeostasis Primarily by Setting the Basal Levels of Hepatic Hepcidin Expression through A Nonproteolytic Mechanism

Caroline A. Enns, Tyler Weiskopf, Richard H. Zhang, Jeffrey Wu, Shall Jue, Makiko Kawaguchi, Hiroaki Kataoka, and An-Sheng Zhang

List of supporting information:

Table S1
Table S2
Table S3
Figure S1
Figure S2

Supplemental **Table S1.** Hematologic parameters for *Spint2*$^{fl/fl}$*;Alb-Cre*$^{+}$ and wild-type *Spint2*$^{fl/fl}$*;Alb-Cre*$^{-}$ littermates.

	Spint2$^{fl/fl}$;Alb-Cre^{-} (male) 5 wks (n=7)	*Spint2*$^{fl/fl}$;Alb-Cre^{+} (male) 5 wks (n=7)	*Spint2*$^{fl/fl}$;Alb-Cre^{-} (female) 5 wks (n=4)	*Spint2*$^{fl/fl}$;Alb-Cre^{+} (female) 5 wks (n=4)	*Spint2*$^{fl/fl}$;Alb-Cre^{-} (male) 8 wks (n=4)	*Spint2*$^{fl/fl}$;Alb-Cre^{+} (male) 8 wks (n=5)	*Spint2*$^{fl/fl}$;Alb-Cre^{-} (female) 8 wks (n=4)	*Spint2*$^{fl/fl}$;Alb-Cre^{+} (female) 8 wks (n=6)
RBC (x10^{6}/µl)	8.46±0.12	8.21±0.78	8.89±0.55	8.84±0.32	8.47±0.11	8.76±0.25	9.15±0.30	8.94±0.57
Hb (g/dL)	12.31±0.20	12.18±1.00	13.57±0.65	13.22±0.25	12.28±0.10	12.56±0.87	13.50±0.66	13.07±0.59
HCT (%)	41.03±1.20	41.52±1.43	44.07±1.16	44.22±1.06	39.10±0.80	40.96±1.71	41.70±1.33	41.73±1.95
MCV (fL)	48.53±1.01	49.77±1.08	49.57±1.35	50.05±1.71	46.12±1.21	46.72±1.15	45.55±0.29	46.75±1.71
MCH (pg)	14.56±0.26	14.84±0.54	15.27±0.37	14.97±0.56	14.50±0.29	14.54±0.63	14.72±0.37	14.63±0.42

RBC, red blood cells; Hb, hemoglobin; HCT, hematocrit; MCV, mean cell volume; MCH, mean corpuscular hemoglobin. Data are expressed as mean ± SD. Two-tailed student-T test was used to analyze the data between wild-type (WT) and *Hai-2*$^{-/-}$ mice at 5 and 8 weeks old for each gender. *, P<0.05; **, P<0.01.

Supplemental **Table S2.** Hematologic parameters for wild-type mice (WT) and *Hai-2*$^{-/-}$ mice fed a control diet (ICD) or iron-deficient diet (IDD)

	Spint2$^{fl/fl}$;Alb-Cre^{-} (male) ICD (n=7)	*Spint2*$^{fl/fl}$;Alb-Cre^{-} (male) IDD (n=8)	*Spint2*$^{fl/fl}$;Alb-Cre^{-} (female) ICD (n=7)	*Spint2*$^{fl/fl}$;Alb-Cre^{-} (female) IDD (n=9)	*Spint2*$^{fl/fl}$;Alb-Cre^{+} (male) ICD (n=13)	*Spint2*$^{fl/fl}$;Alb-Cre^{+} (male) IDD (n=13)	*Spint2*$^{fl/fl}$;Alb-Cre^{+} (female) ICD (n=9)	*Spint2*$^{fl/fl}$;Alb-Cre^{+} (female) IDD (n=8)
RBC (x10^{6}/µl)	9.28±0.35	9.38±0.24	9.11±0.36	8.92±0.42	8.78±0.45	8.84±0.44	9.07±0.39	9.18±0.43
Hb (g/dL)	14.23±0.28	13.23±0.71*	13.87±0.41	12.47±0.65	13.72±0.78	12.86±0.82*	13.88±0.66	12.94±1.61
HCT (%)	44.97±0.33	43.36±1.02*	45.07±1.58	42.17±1.99	42.47±3.37	41.42±1.97	44.64±1.52	42.51±3.11
MCV (fL)	48.50±0.52	46.76±1.24	49.47±1.46	47.27.05±0.42	48.31±2.23	46.90±0.85*	49.24±1.83	47.21±2.26
MCH (pg)	15.37±0.38	14.24±0.49**	15.23±0.57	13.97±0.93	15.62±0.35	14.57±0.81**	15.29±0.51	14.09±1.67

RBC, red blood cells; Hb, hemoglobin; HCT, hematocrit; MCV, mean cell volume; MCH, mean corpuscular hemoglobin. Data are expressed as mean ± SD. Two-tailed student-T test was used to analyze the data between ICD and IDD groups of wild-type (WT) and *Hai-2*$^{-/-}$ mice for each gender. *, P<0.05; **, P<0.01.

***Supplemental* Table S3. List of mouse-specific primers used for qRT-PCR analysis**

Gene	Forward primer	Reverse primer
β-actin	5'-CTGCCTGACGGCCAGGT-3'	5'-TGGATGCCACAGGATTCCAT-3'
Bmp6	5'-AGCACAGAGACTCTGACCTATTTTTG-3'	5'-CCACAGATTGCTAGTTGCTGTGA-3'
Hepcidin	5'-CACCAACTTCCCCATCTGCATCTT-3'	5'-GAGGGGCTGCAGGGGTGTAGAG-3'
Hfe	5'-TCTGGGACAGCAAGTGCCTAC-3'	5'-GGCATCCAGTGGTTGGTTGT-3'
Hjv	5'-ATCCCCATGTGCGCAGTTT-3'	5'-GCTGGTGGCCTGGACAAA-3'
Id1	5'-ACCCTGAACGGCGAGATCA-3'	5'-TCGTCGGCTGGAACACATG-3'
IL-6	5'-TTCCATCCAGTTGCCTTCTTG-3'	5'-TTGGGAGTGGTATCCTCTGTGA-3'
Neo1	5'-CGAGCTGCGTGCAAATCA-3'	5'-ATCCATAGGTCTGGAGGCTTCAC-3'
Spint1	5'-GCTGTGCCCGATTCACCTATG-3'	5'-GACCACTATGATGCAGATGACCAGA-3'
Spint2	5'-TATGGAGGCTGTGAAGGCAAT-3'	5'-AGAGTCGGCTCCATTCCTGTT-3'
Tfr2	5'-GCTGGGACGGCGGTGACTT-3'	5'-GAGTTGTCCAGGCTCACGTACA-3'
Tmprss6	5'-TTGCTGGTCTTGGCTGCGCT-3'	5'-AATGACGGTTGAGCACCCGGAG-3'

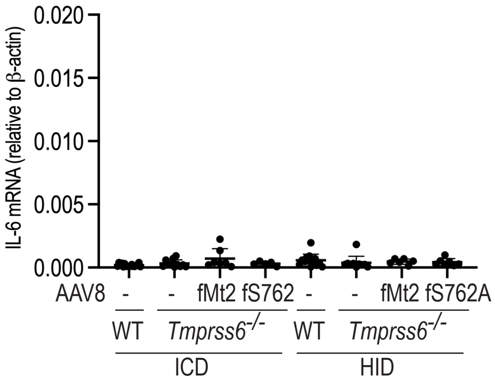

Supplemental Figure S1. qRT-PCR analysis of hepatic IL-6 mRNA in the mice as described in **Figure 4.** Results are expressed as the amount relative to that of ß-actin for each sample. Data are expressed as mean ± SD. One-way ANOVA and Tukey's post-test were used to analyze the data relative to the WT-Tmprss6+/+/ICD group. No statistical significance was noticed.

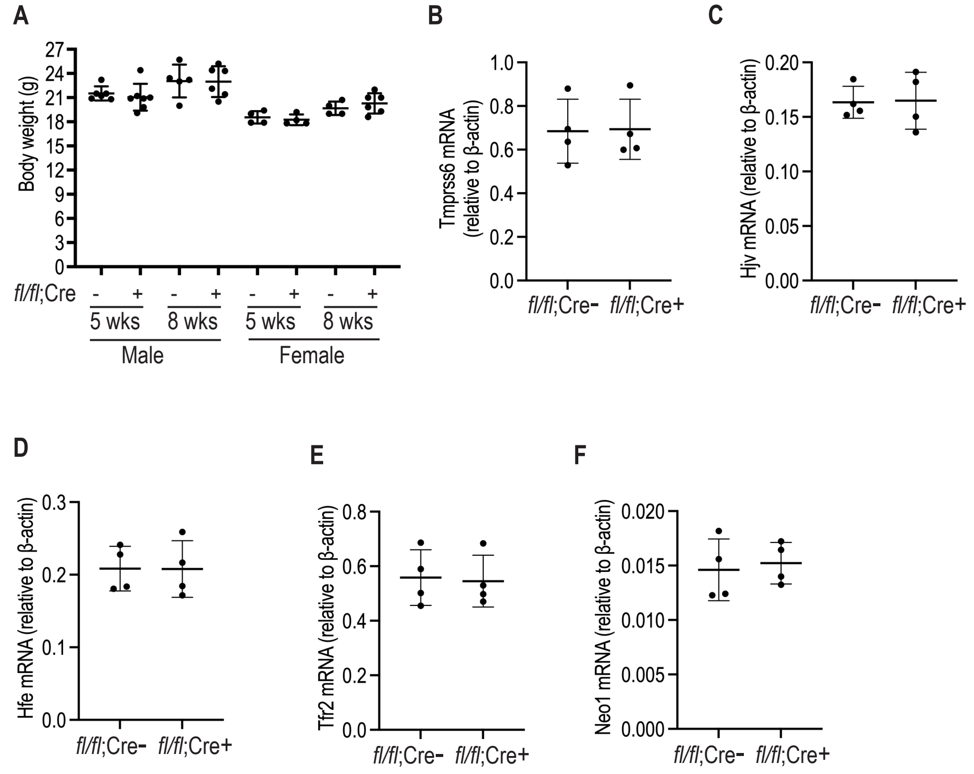

Supplemental Figure S2. **A)** Body weight of 5 and 8-week-old male and female *Spint2*$^{fl/fl}$;Alb-Cre- and *Spint2*$^{fl/fl}$;Alb-Cre+ mice. **B-F)** qRT-PCR analysis of Tmprss6, Hjv, Hfe, Tfr2, and Neo1 mRNA in isolated hepatocytes from 8-week-old *Spint2*$^{fl/fl}$;Alb-Cre- and *Spint2*$^{fl/fl}$;Alb-Cre+ mice as described in the legends to **Figure 6A**. All qRT-PCR results are expressed as the amount relative to that of β-actin for each sample. The mean values and the standard deviation (SD) are presented. Two-tailed Student t test was used to analyze the data for each gender and age-matched group. No statistical significance was noticed.
